# SARS-CoV-2 paediatric chest X-ray findings during the Omicron variant wave

**DOI:** 10.4102/sajid.v40i1.768

**Published:** 2025-10-29

**Authors:** Javeria Hussain, Linda T. Hlabangana, Nasreen Mahomed, Gary Reubenson, Sharadini K. Gounden, Ashesh I. Ranchod

**Affiliations:** 1 Department of Diagnostic Radiology, Faculty of Health Sciences, University of the Witwatersrand, Johannesburg, South Africa; 2 Department of Paediatrics and Child Health, Faculty of Health Sciences, University of the Witwatersrand, Johannesburg, South Africa; 3 Department of Diagnostic Radiology, Faculty of Health Sciences, Helen Joseph Hospital, Johannesburg, South Africa

**Keywords:** coronavirus, COVID-19, Omicron, paediatric, chest X-ray, chest radiograph

## Abstract

**Background:**

The coronavirus disease 2019 (COVID-19) is caused by a novel beta coronavirus (severe acute respiratory syndrome coronavirus 2 [SARS-CoV-2]). The Omicron variant was first identified in November 2021 in multiple countries, including South Africa. The authors aimed to assess chest radiographs to ascertain whether unique radiographic manifestations were related to this variant.

**Objectives:**

The primary objective was to identify key chest X-ray findings in the South African paediatric population testing positive for SARS-CoV-2 during the Omicron era. The secondary objective was to help differentiate between Omicron chest X-ray findings and published findings regarding all preceding variants.

**Method:**

A retrospective cohort review was conducted at three main healthcare academic centres, in which 94 paediatric chest X-rays were assessed by three consultant radiologists to identify key imaging findings.

**Results:**

Ground-glass opacities were more common among infants (45.4%) than children (20%) and adolescents (20%; *p* = 0.001). Peribronchial thickening was high in all age groups: 97.7% in infants, 87.5% in children and 70% in adolescents (*p* = 0.019). Consolidation was seen in 28.6% of infants, 19.1% of children and 33.3% of adolescents (*p* = 0.065). Diffuse disease (involving all lobes) was seen in 54.5% of infants, 52.5% of children and 10% of adolescents (*p* = 0.033).

**Conclusion:**

Infants had the most chest X-ray findings, with peribronchial thickening followed by ground-glass opacities being the most common.

**Contribution:**

The findings suggest difference neither between Omicron and preceding waves nor between this study and previously published data.

## Introduction

Coronavirus disease 2019 (COVID-19) is caused by a novel beta coronavirus, severe acute respiratory syndrome coronavirus 2 (SARS-CoV-2). The virus was first identified during an outbreak in Wuhan, China, in December 2019.^1^ The World Health Organization (WHO) declared COVID-19 a Public Health Emergency of International Concern on 30 January 2020.^2^ The Omicron variant was first identified in November 2021 in South Africa and Botswana and then in multiple countries.^3^ Previously circulating variants included the Beta variant (B.1.351), first identified in South Africa in October 2020; the Alpha variant (B.1.1.7), first identified in the United Kingdom in December 2020; the Delta variant (B.1.617.2), first identified in India in December 2020 and the Gamma variant (P.1), first identified in Brazil in December 2020.^4^

During the first three waves of the COVID-19 epidemic in South Africa, clinical manifestations ranged from mild to moderate in most children.^3,5,6^ However, an increase in child hospitalisations in the Gauteng province in November 2021 raised concern.^3^ With the presence of the new Omicron variant and the concurrent rise in paediatric hospital admissions, the authors aimed to assess the chest radiographs to determine whether unique radiographic manifestations were related to this variant. Although significant literature has been published regarding chest imaging in COVID-19, studies focusing on the paediatric population – and particularly on the Omicron variant – remain limited. The Omicron variant marked the fourth wave of the epidemic in South Africa,^3^ during which paediatric cases exceeded adult cases and paediatric admissions exceeded those during previous waves.^3^

The clinical spectrum of COVID-19 ranges from being asymptomatic or mild flu-like illness to severe respiratory failure, which may result in death. Common respiratory manifestations include viral pneumonia, with necrotising pneumonia being a severe complication.^7^ COVID-19-related laryngotracheobronchitis has been associated with the Omicron variant.^7^ Adult respiratory distress syndrome (ARDS) was less commonly reported in children with severe COVID-19 than in adults.^7^ Bronchopneumonia may occur at any stage of the illness, whereas organising pneumonia typically arises later, often because of superimposed infections.^8^ The current literature supports that COVID-19 clinical and radiological features vary by age group.^9,10^ Symptomatic patients were more likely to demonstrate a bilateral diffuse interstitial pattern on imaging.^11^ Ground-glass opacification and consolidation are common findings on computed tomography (CT) scans. Although peribronchial thickening is frequently observed, it is considered a subjective sign with high inter-observer variability.^12^ A rare but severe complication of fatal pneumothorax was documented in an infant with the Omicron variant.^13^ Bronchial thickening was seen more commonly in younger children than in adults, and ground-glass opacities (GGOs) were more common in older children.^14,15^ Ground-glass opacities were also associated with increased hospitalisation risk.^15^ Notably, hyperinflation, a common finding in other viral infections, was not observed in COVID-19.^16,17^ ‘Crazy paving’ and the halo sign can also be seen, although these are more commonly CT findings.^15^

Early in the pandemic, Foust et al.^15^ divided imaging characteristics into four categories: typical, indeterminate, atypical and negative. Typical findings were bilateral, peripheral or subpleural with GGOs or consolidation. Indeterminate findings were unilateral peripheral or peripheral with central GGOs or peribronchial thickening. Atypical findings were unilateral consolidation, central bilateral GGOs, pleural effusions and lymphadenopathy. Lastly, the negative category had no abnormal chest radiograph findings. While children had fewer clinical and radiological features than adults, bronchial thickening was more frequently observed in children (28.6% in children versus 2.3% in adults).^16^ Pleural effusion and interstitial opacities were commonly seen in children with Multisystem Inflammatory Syndrome in Children (MIS-C).^17,18^ In a multicentre literature review of 850 COVID-19 paediatric patients, the disease course was mild, with predominant imaging findings of ground-glass opacification and consolidation (61.5% of patients), in a peripheral and lower-lobe distribution.^19^

Although the radiological manifestations of COVID-19 are nonspecific, chest X-rays can neither establish a diagnosis nor serve as a screening tool for paediatric COVID-19. However, they could potentially guide care. Many children admitted with COVID-19 would not routinely be imaged with radiographs or chest CT.

## Research methods and design

A retrospective cohort review was conducted at three main healthcare academic centres in a metropolitan city in South Africa. Institutional review board approval was obtained from each centre prior to study initiation. Inclusion criteria were paediatric patients aged between 1 month and 12 years who tested positive for SARS-CoV-2 and had chest X-rays between 1 November 2021 and 30 November 2022. This period was the peak of the Omicron wave. Neonates (children < 1 month of age) were excluded, as this subset of patients is predisposed to severe lung pathologies (e.g. respiratory distress syndrome, meconium aspiration syndrome, congenital pneumonia and bronchopulmonary dysplasia), and these conditions may present with similar chest X-ray findings to COVID-19. Additionally, patients over 12 years old were excluded, as this is the cut-off age for paediatric services at most study sites.

Data and patient information meeting the inclusion criteria were obtained from the DATCOV database, identifying 492 patients with SARS-CoV-2 PCR-positive nasopharyngeal swabs. A search of the relevant departments’ Picture Archiving and Communication System (PACS) was conducted to retrieve corresponding chest X-rays of the identified patients. Ninety-four patients with available chest X-rays were included in the study. These images were saved in Digital Imaging and Communications in Medicine (DICOM) format and distributed among the three experienced specialist radiologists. Two readers have over 20 years of radiology experience, and the third has 10 years of experience; all have an interest in paediatric imaging. Each reader independently reviewed all 94 X-rays, with findings determined by majority consensus.

Basic demographic patient data were collected (e.g. age, comorbidities, indication for study, study date and other relevant lab results). Each reader evaluated and reported on every X-ray for its pathological findings and distribution of disease on the chest X-rays. The type of X-ray was noted (i.e. frontal, lateral or both). Imaging findings, such as GGOs, consolidation, peribronchial thickening or pleural effusions, were documented. The lobes involved were also documented, either single lobe or all lobes. The latter was reported as diffuse disease. The findings from each reader were anonymised and tabulated on a spreadsheet (see Online Appendix 1 for template). The majority findings between the three readers were documented. The data were analysed using IBM SPSS Statistics version 30. Descriptive statistics were used to analyse the patients’ demographic and clinical characteristics, expressed as counts and percentages. The X-ray findings were compared with the demographic and clinical factors using the Pearson chi-square and Fisher’s exact tests. Inter-rater reliability in the reading of the X-rays was examined through the Fleiss kappa. All statistical analyses were considered significant for *p*-values < 0.05.

### Ethical considerations

An application for full ethical approval was made to the Human Research Ethics Committee (Medical) – University of the Witwatersrand, and ethics consent was received on 25 March 2024. The ethics approval number is M230404.

## Results

Ninety-four (54 male patients – 57.4% and 40 female patients – 42.6%) patients with X-rays were evaluated. X-rays were matched to the time of the positive SARS-CoV-2 result. Forty-four patients (46.8%) were symptomatic for COVID-19 with respiratory symptoms including cough, dyspnoea and respiratory distress. Twenty-nine patients (30.8%) were asymptomatic for respiratory infection and had incidental positive SARS-CoV-2 results. Twenty-one files could not be traced (22.3%). Ages ranged from 1 month to 12 years. The median age of the patients was 1 year, with an interquartile range of 4 years 8 months. Patients were further divided based on age; these categories included infants (1–11 months), children (1–9 years) and adolescents (> 10 years). Based on these ranges, 44 (46.8%) patients were infants, 40 (42.6%) were children and 10 (10.6%) were adolescents.

Patient comorbidities are presented in Table 1. HIV was the most common comorbidity, accounting for eight (8.5%) patients. Six children had underlying malignancy (i.e. Burkitt lymphoma, T-cell non-Hodgkin lymphoma, osteoblastic osteosarcoma and acute lymphoblastic leukaemia). Five children had underlying congenital cardiac diseases (i.e. patent ductus arteriosus, ventricular septal defect, atrio-ventricular canal defect and tetralogy of Fallot). Tuberculosis (TB) co-infection was seen in two patients, one with pulmonary TB and one with TB of the spine. Of note, one child had a pericardial effusion with the pericardial fluid testing positive for SARS-CoV-2. Four children had concurrent respiratory viruses: rhinovirus,^2^ adenovirus^1^ and human bocavirus.^1^ Two children had concurrent bacterial infections: one with extremely drug-resistant *Acinetobacter baumannii* and carbapenem-resistant Enterobacterales and one with extended spectrum beta-lactamase-producing *Klebsiella bacteraemia*. Thirty-seven (39.4%) patients had both anteroposterior (AP) and lateral views of the chest X-ray, while 57 (60.6%) had only a frontal view available.

**TABLE 1:** Patient co-morbidities.

Comorbidity	*n*	%
HIV	8	8.5
Tuberculosis	2	2.1
Bacteraemia	7	7.4
Diabetes	9	9.5
Cardiac disease	5	5.3
Malignancy	6	6.4

**FIGURE 1:**
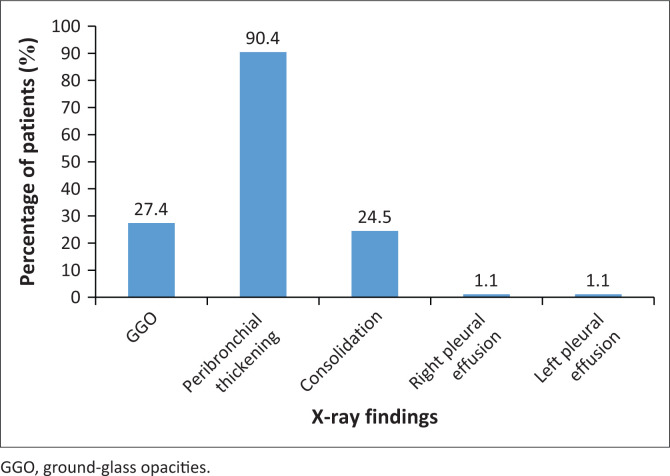
Comparison of X-ray findings.

**FIGURE 2:**
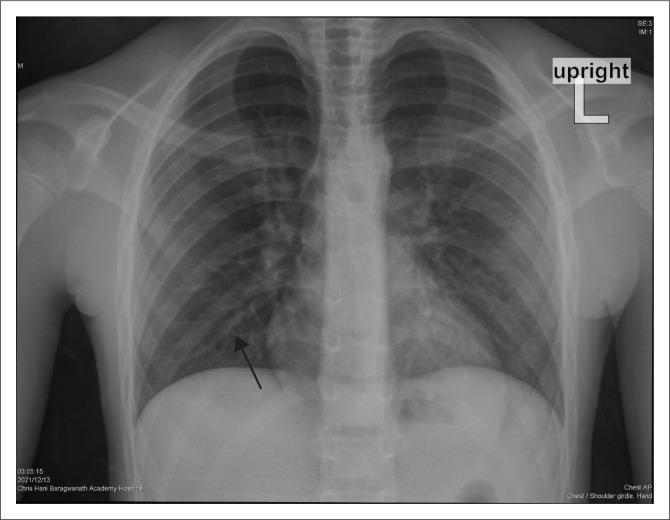
Frontal chest radiograph showing peribronchial thickening of the right lower lobe bronchus (black arrow) in a 9-year-old male patient with no comorbidities. The peribronchial thickening is seen bilaterally, implying widespread inflammation of the airways.

Overall, X-ray findings reported among the readers included GGOs, peribronchial thickening, consolidation and pleural effusions (see Figure 1). Peribronchial thickening (see Figure 2) was the most common finding, being present in 85 (90.4%) patients. Eight chest X-rays were reported as normal.

**FIGURE 3:**
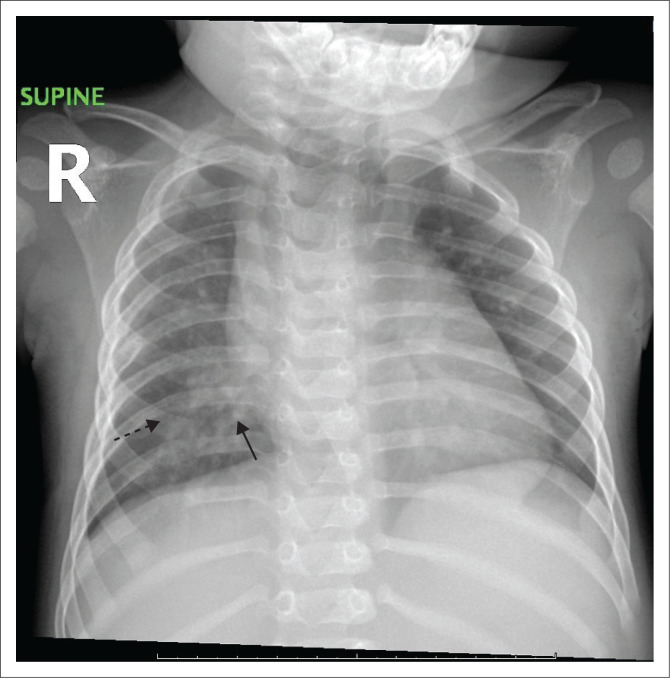
Supine radiograph of an infant showing peribronchial thickening (solid black arrow) and ground-glass opacities (dashed black arrow) involving all lung lobes, indicating diffuse disease.

When looking at analysis by age, GGOs were more common among infants (45.4%) than children (20%) and adolescents (20%; *p* = 0.001). Peribronchial thickening was high in all age groups: 97.7% in infants, 87.5% in children and 70% in adolescents (*p* = 0.019). Consolidation was seen in 28.6% of infants, 19.1% of children and 33.3% of adolescents (*p* = 0.065). Diffuse disease (involving all lobes; see Figure 3) was evident in 54.5% of infants, 52.5% of children and 10% of adolescents (*p* = 0.033). Lung findings between male patients and female patients were similar.

Readers further evaluated the anatomical distribution of lung involvement (Table 2). The findings demonstrated no clear predilection for any specific pulmonary lobe. Overall, 46 patients (48.9%) were found to have diffuse lung involvement.

**TABLE 2:** Region of the lung involved.

Anatomical region of the lung	*n*	%
Left upper lobe	35	37.2
Left lower lobe	28	29.8
Right upper lobe	38	40.4
Right middle lobe	30	31.9
Right lower lobe	35	37.2

## Discussion

In the preceding COVID-19 variant waves, admission of paediatric patients was relatively low.^1^ During the Omicron variant, presentation and hospitalisations in children were higher.^1^ This increase in case numbers, particularly in Gauteng, along with S-gene target failure (SGTF) on polymerase chain reaction (PCR) testing, was previously seen in the alpha variant, and this prompted genomic testing, which confirmed that the dominant variant at the time was Omicron.^20^ Using the published data from GISAID, which tracked the relative variant frequency in Africa between November 2021 and November 2022, Omicron was shown to be the most frequently isolated variant (reaching up to 98.9%).^20^ This finding may relate to the higher transmissibility of Omicron, vaccination programmes targeted at adults and greater transmission among school-going children.^21^

At the time of the study, vaccination programmes did not routinely include children over 12 years old. Chest imaging is not routinely performed for screening in patients with COVID-19. According to the American College of Radiology (ACR), chest radiography or CT cannot diagnose SARS-Cov-2, nor can imaging differentiate COVID-19 from other causes of viral and bacterial pneumonia.^19^ Instead, it is used to assess for complications of COVID-19 such as abscess, pleural effusion or bronchopleural fistula.^19^

**FIGURE 4:**
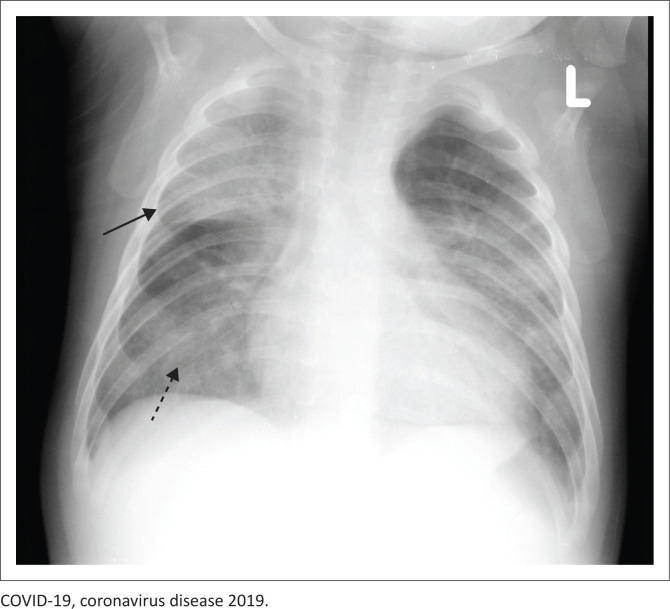
Frontal radiograph of a 4-month-old male patient showing consolidation of the right upper lobe (solid black arrow) and ground-glass opacities (dashed black arrow) in bilateral lung fields. This infant had COVID-19 with a superimposed bacterial infection.

Ground-glass opacities (see Figure 4) – a term frequently associated with CT imaging – refer to increased attenuation in the lung. This term can also be applied to chest radiography and refers to an ill-defined, hazy airspace opacity without an air bronchogram through which pulmonary vessels are visible, differentiating it from consolidation.^22^ Histologically, GGOs result from partial filling of alveoli (with blood, pus, water or cells), and alveoli walls are thickened or collapsed (atelectasis). Consolidation is a dense airspace opacity with air bronchograms.^22^ Histologically, it is because of the complete filling of the alveoli with substances such as blood, pus, water or cells. This is seen in pneumonia, aspiration, haemorrhage, ARDS or pulmonary oedema in the acute phase.

Peribronchial thickening is abnormal bronchial wall thickening because of infection, inflammation or neoplasm.^22^ This study demonstrates a higher percentage of peribronchial thickening than previous studies (see Table 3). Ground-glass opacities (correlating with airspace opacities), while comparatively lower than peribronchial thickening, also had a lower percentage than published reports. An unusual finding was that consolidation (as a radiographic finding) was higher than in other studies, and that pleural effusions may be a complication of the disease.

**TABLE 3:** Comparison of chest X-ray findings with data from preceding coronavirus disease 2019 variants.

Reference	Country of study	Number of patients	Variant	Percentage			
				GGO	Peribronchial thickening	Consolidation	Distribution
Our study	South Africa	94	Omicron	27.7	94.4	24.5	48.9‡
Nino et al.^10^	United States	95	Original strain and beta variant	35.0	34.0	Grouped with GGOs	Not given
Das et al.^14^	United Arab Emirates	56	Original strain and beta variant	45.4	0.0	54.0†	Not given
Vendhan et al.^14^	South India	90	Original strain and beta variant, study partly conducted during the Omicron variant	2.0	68.0	11.0	69.0§ 31.0‡

Note: Please see full reference list of this article: Hussain J, Hlabangana LT, Mahomed N, Reubenson G, Gounden SK, Ranchod AI. SARS-CoV-2 paediatric chest X-ray findings during the Omicron variant wave. S Afr J Infect Dis. 2025;40(1), a768. https://doi.org/10.4102/sajid.v40i1.768 for more information.

GGO, ground-glass opacities.

† , Combined with GGO;

‡ , Diffuse disease;

§ , Perihilar distribution.

The WHO declared an end to the global health emergency on 05 May 2023. Despite the decreasing interest in COVID-19 and many studies on the imaging findings in COVID-19 in adults in South Africa, there has been limited research on paediatric cases and their imaging findings, especially during the Omicron wave. Lin et al.^23^ studied CT findings of 37 children infected with Omicron in China and found 70% of patients had negative chest CT findings. Positive findings were GGOs, consolidation or both; no predominant lung distribution was noted.

### Limitations

The limitations of this study were its retrospective design, lack of a comparator group from preceding waves and limited access to patient clinical data and record-keeping at these hospitals, which prevented monitoring of patient outcomes and progress. In addition, because children do not routinely get X-rays in COVID-19^24^, those imaged were mostly inpatients and represent severely ill patients. Many images were also lost or untraceable, resulting in a limited sample size. A larger sample size could have led to a more robust study.

## Conclusion

Infants exhibited the highest number of chest X-ray abnormalities, with peribronchial thickening being the most common finding, followed by GGOs. Children (aged 1–10 years) demonstrated a comparable frequency of lung changes, while adolescents showed the fewest abnormalities. Diffuse disease was observed more in infants and young children than in adolescents. Overall, the imaging findings suggest no difference between Omicron and preceding waves or between this study and previously published data. COVID-19 remains a matter of interest as the virus evolves, similarly changing its clinical and radiographic manifestations. Familiarity with the imaging findings is essential to broaden differential diagnoses beyond common viral or bacterial aetiologies of pneumonia.
